# Navigating medicine and justice: A life history and phenomenological study of forensic doctors' experiences

**DOI:** 10.1111/1556-4029.70089

**Published:** 2025-05-19

**Authors:** Mateus Eduardo Romão, Ilaria Setti, Gill Thomson, Giorgia Alfano, Serena Barello

**Affiliations:** ^1^ Department of Brain and Behavioral Sciences University of Pavia Pavia Italy; ^2^ Maternal, Parental and Infant Nutrition & Nurture (MAINN) University of Central Lancashire Preston Lancashire UK; ^3^ Unit of Applied Psychology, IRCCS Mondino Foundation Pavia Lombardia Italy

**Keywords:** coping strategies, forensic medicine, interpretative phenomenological analysis, life histories, professional identity, psychological distress, qualitative research

## Abstract

Forensic doctors play a crucial role at the intersection of medicine and law, offering expertise in legal matters. However, their work exposes them to significant emotional and psychological stress, increasing the risk of burnout. Despite the critical nature of their profession, little research has explored how forensic doctors construct their professional identities, cope with work‐related challenges, and make sense of their professional experiences. To address this gap, we adopted a life‐history approach combined with interpretative phenomenological analysis to examine the subjective career trajectories and meaning‐making processes of forensic doctors in Italy. Semi‐structured interviews were conducted with 12 participants, capturing their personal and professional histories. An in‐depth analysis identified five key themes: (1) the nature of forensic medicine—participants used metaphors to describe the complexity, artistry, and investigative nature of their work; (2) the professional identity of forensic doctors—how they develop a sense of duty, ethical commitment, and professional resilience throughout their careers; (3) core resources needed to be a forensic doctor—the role of mentorship, specialized skills, and coping strategies in sustaining their careers; (4) what does death have to teach?—the personal impact of continuous exposure to death, shaping their perspectives on life and mortality; (5) esse cum—the challenges, stigma, and stereotypes forensic doctors face in their interactions with colleagues, legal professionals, and society. This study explores the professional identities and coping mechanisms of forensic doctors, revealing that despite facing stigma, emotional strain, and burnout, they find meaning in their work, emphasizing justice and resilience.

**Highlights**

- This is the first qualitative study using the life‐history approach to explore the lived experiences and personal stories of a purposive sample of 12 Italian forensic doctors.
- Provides an in‐depth, idiographic exploration of professional trajectories rather than a representative overview of the Italian forensic doctor population.
- Identifies five key themes that illustrate career challenges, coping mechanisms, and the impact of societal stigma.
- Demonstrates how continuous exposure to death shapes professional identity, life perspectives, and emotional resilience.
- Offers preliminary insights to inform interventions aimed at reducing stigma, strengthening mentorship, and improving communication between forensic doctors and stakeholders.

## 1 INTRODUCTION

Forensic doctors, also known as forensic pathologists, play a crucial role at the intersection of medicine and the legal system. Their responsibilities include performing autopsies, examining crime scenes, evaluating trauma, and providing expert testimony in court [1, 2]. This position requires them to bridge the gap between medical findings and legal implications, making their expertise vital in resolving criminal cases, administering justice, and protecting public safety [1, 2]. Forensic doctors' unique position within the justice system likely shapes a distinct professional identity—a critical aspect of how individuals perceive themselves within their occupational role—perhaps characterized by a strong sense of duty and ethical commitment [3, 4]. Investigating this could provide valuable insights into what drives these professionals and how they view their contributions to society [3, 4].

How forensic doctors cope with the negative impacts of their role is important, as the well‐being of practitioners directly influences the quality and reliability of their outputs [2, 3]. Evidence highlights that the emotional and psychological burden of their work can lead to significant mental health issues, including stress, burnout [5], and vicarious trauma [1]. The high stakes of providing accurate and reliable medical opinions further exacerbate the work‐related stress, as errors can have profound legal and ethical consequences [2, 3]. Forensic doctors may adopt various stress management strategies, including peer support, professional supervision, and personal resilience techniques, such as compartmentalization and mindfulness [3, 4]. Identifying and understanding these coping mechanisms may help in the development of better support programs that bolster the emotional and psychological resilience of forensic doctors, thereby enhancing their professional efficacy and personal well‐being [3, 4].

Despite the uniqueness of the forensic work setting, the professional experiences of forensic doctors remain underexplored in the scientific community. To address this research gap, this study aimed to explore the lived experiences and life histories of forensic doctors by employing a life‐history approach using interpretative phenomenological analysis (IPA). By capturing the lived experiences of their role, this research sought to provide an in‐depth understanding of their professional identity, the challenges they encounter, their coping mechanisms, and how they perceive the societal views on their profession.

## 2 METHODS

### 2.1 Research design

This study employed a qualitative life‐history approach using IPA to explore the lived experiences of forensic doctors. The life‐history approach is a well‐established method in qualitative research that examines individual career trajectories and identity development over time, often in response to changing professional demands and personal experiences [6, 7]. This approach is particularly valuable for professions characterized by high emotional demands, ethical dilemmas, and evolving personal identities, such as forensic medicine [8].

IPA was chosen because it enables in‐depth exploration of personal experiences, focusing on how individuals make sense of their professional realities [9]. Unlike quantitative approaches that prioritize breadth and statistical generalizability, IPA and the life‐history approach emphasize depth, subjective meaning‐making, and career narratives as they unfold over time [10] This methodological combination allowed us to examine how forensic doctors construct their professional identities, cope with stress, and navigate societal perceptions of their work. IPA is a robust and well‐established methodology specifically designed to examine how individuals make sense of their personal and professional experiences. Unlike quantitative approaches that prioritize breadth and statistical generalizability, IPA focuses on depth and the nuanced understanding of subjective experiences within a specific context. It emphasizes the unique perspectives of individuals, enabling researchers to uncover the underlying meanings and interpretations of their lived realities. This idiographic approach is particularly suited for exploring complex phenomena, such as the professional challenges and coping mechanisms of forensic doctors, as it allows for a detailed and rich analysis of each participant's narrative. By prioritizing individual experiences, IPA provides insights that can inform practice and policy, complementing the broader generalizations of quantitative research [11].

### 2.2 Participants

Participants were recruited using non‐probabilistic, purposeful sampling, a widely accepted approach in life‐history research and qualitative inquiry [6, 12, 13] Recruitment, based on the research team's professional network, aimed to include individuals with diverse backgrounds in terms of gender, years of experience, and professional trajectories, ensuring a broad range of perspectives [11]. Inclusion criteria required participants to have regular exposure to death‐related scenarios and fluency in Italian.

The goal was not statistical representativity but rather to collect their life histories, capturing rich, in‐depth narratives on forensic medical practice. Given the depth‐oriented nature of IPA and the life‐history approach, small sample sizes are standard, enabling detailed, case‐by‐case exploration of lived experiences [13]. In particular, the sample size was determined according to IPA methodological guidelines, which recommend 4–10 participants for idiographic, life‐history research, prioritizing detailed personal narratives over broad generalizability [14].

The final sample consisted of 12 forensic doctors, selected to reflect varied professional experiences, gender perspectives, and career stages. This aligns with best practices in qualitative research, where small but information‐rich samples provide deep insights into professional identity formation and longitudinal career development [13, 14].

### 2.3 Ethical considerations

The study was conducted following the Helsinki guidelines for human research. The protocol of the study was approved by the ethical committee (protocol no: 176/24) of the Department of Brain and Behavioral Sciences from the University of Pavia, Italy. All participants signed informed written consent forms before data collection.

### 2.4 Data collection

Data were collected through semi‐structured interviews conducted by researchers with training in psychology and extensive experience in qualitative methods. The interviews, conducted in Italian, were designed to elicit in‐depth narratives, focusing on participants' experiences, perceptions, and reflections related to their work as forensic doctors. The interview guide included open‐ended questions, enabling participants to share their stories freely while also addressing specific topics, such as professional identity, challenges, coping strategies, and societal perceptions (Table 1).

**TABLE 1:** Interview guide for identifying professional identity, challenges, coping strategies, and societal perceptions.

**To begin with, I would like to ask you to tell me about your professional experience…**.
If you had to describe your job with an image, a metaphor… what would you choose? Why? Can you tell me about an episode related to your work experience that you consider particularly significant and that has remained in your memory?
**Thinking about how you started this job…**
What motivated you to pursue this career?
**And thinking about today…**
What keeps you connected/involved in your work despite the challenges you might encounter in dealing with a delicate topic like suffering and death on a daily basis? How do you manage the emotional pain and stress associated with your job? Are there strategies or resources that you find particularly useful? How would you describe your relationship with your colleagues? In your experience, is bureaucracy something stressful and challenging? Why?
**Let's now talk about how one can prepare to work in your field…**
What skills or personal qualities do you consider essential for working successfully in your field? What risks do you think there are for those who wish to pursue a profession that brings workers into daily contact with the theme of suffering and death? Can you give examples of situations or scenarios that you consider potentially risky? Do you think specific training on coping strategies or emotion management would be useful for workers in your field?
**And to conclude…**
How do you think your professional experience has influenced your view of life and death in general? What would you like the public/society to better understand about your work and the topic of death? What are your hopes and ambitions for the future of your work and the sector in general? What would you like to see change or improve? Is there anything else you would like to share?

Interviews were held in private and comfortable settings to ensure confidentiality and foster an environment conducive to open and honest discussions. Each interview lasted c. 60–90 mins and was audio‐recorded with participants' consent. To ensure accurate and reliable translation, two bilingual researchers independently translated and back‐translated the participants' quotations from Italian to English.

### 2.5 Data analysis

The life‐history approach and IPA were employed to analyze the transcribed interviews, capturing forensic doctors' career trajectories, meaning‐making processes, and coping mechanisms over time. The life‐history approach focuses on how individuals construct and interpret their professional experiences, while IPA emphasizes an in‐depth, idiographic exploration of personal narratives [11].

The analysis process followed the guidelines outlined for the IPA method [15] combined with principles from life‐history research, ensuring a systematic and iterative approach to data interpretation. To enhance trustworthiness and credibility, at least two independent researchers conducted the analysis, ensuring rigorous examination of each participant's life history while acknowledging the subjective meaning‐making process inherent to qualitative research.

The analysis involved the following steps:

1. Familiarization: Researchers independently read and re‐read transcripts to immerse themselves in the data, identifying key moments in participants' professional trajectories and experiences.
2. Initial coding: Significant statements, phrases, and concepts were identified, with a focus on career development, identity formation, and coping strategies in forensic practice.
3. Developing emergent Themes: Initial codes were grouped into broader themes that reflected how forensic doctors construct meaning in their work and navigate professional challenges over time.
4. Searching for connections: The researchers explored relationships among emergent themes, identifying patterns in career trajectories, resilience strategies, and interactions with the legal and medical systems.
5. Moving to the next case: Each participant's life history was analyzed independently, ensuring that personal narratives were preserved before drawing cross‐case connections.
6. Looking for patterns across cases: Finally, the researchers examined commonalities and divergences across participants, identifying shared themes while maintaining an idiographic focus on individual career experiences.

By integrating IPA's in‐depth, idiographic analysis with the narrative structure of the life‐history approach, this study provides a nuanced understanding of how forensic doctors interpret their roles, cope with stress, and navigate their evolving professional identities.

### 2.6 Trustworthiness and credibility

To ensure the trustworthiness and credibility of the findings, the researchers employed several strategies:

1. *Reflexivity:* The researchers engaged in ongoing reflexivity throughout the research process, acknowledging their own biases, assumptions, and preconceptions, and how these might influence the interpretation of the data.
2. *Peer debriefing:* The researchers engaged in regular discussions with peers and colleagues to challenge their interpretations, gain alternative perspectives, and refine the analysis.
3. *Member checking:* The researchers shared the preliminary findings with the participants to ensure that their experiences were accurately represented and to gather feedback on the interpretations.
4. *In‐depth description:* The researchers provided rich and detailed descriptions of the participants' experiences and the research context, allowing readers to assess the transferability of the findings to other settings.

While IPA and the life‐history approach do not traditionally emphasize inter‐rater reliability in the same way as quantitative methodologies, their rigor is ensured through methodological transparency, reflexivity, and collaborative analysis. The life‐history approach, in particular, values the subjective meaning‐making process, recognizing that researcher interpretation is an integral part of understanding how individuals construct their professional narratives. By adhering to these methodological principles and engaging at least two independent researchers, we strengthened analytical robustness, minimizing subjective bias, and enhancing credibility. This collaborative validation process ensures the trustworthiness of the findings, aligning with best practices in qualitative research that prioritize depth, reflexivity, and methodological transparency.

## 3 RESULTS

Twelve Italian forensic doctors participated in the study (Table 2). The sample included four males, with a mean professional experience of 12.25 years and an average age of 40.25 years. Eight females (66.67% of the sample) participated, with a mean professional experience of 9.63 years and an average age of 36.88 years. Detailed participant characteristics are provided in Table 1.

**TABLE 2:** Participant characteristics.

ID participant	Gender	Years of professional experience
P01	Male	1
P02	Female	3
P03	Female	25
P04	Male	5
P05	Female	5
P06	Male	38
P07	Female	19
P08	Female	5
P09	Female	14
P10	Female	4
P11	Female	6
P12	Male	5

**FIGURE 1:**
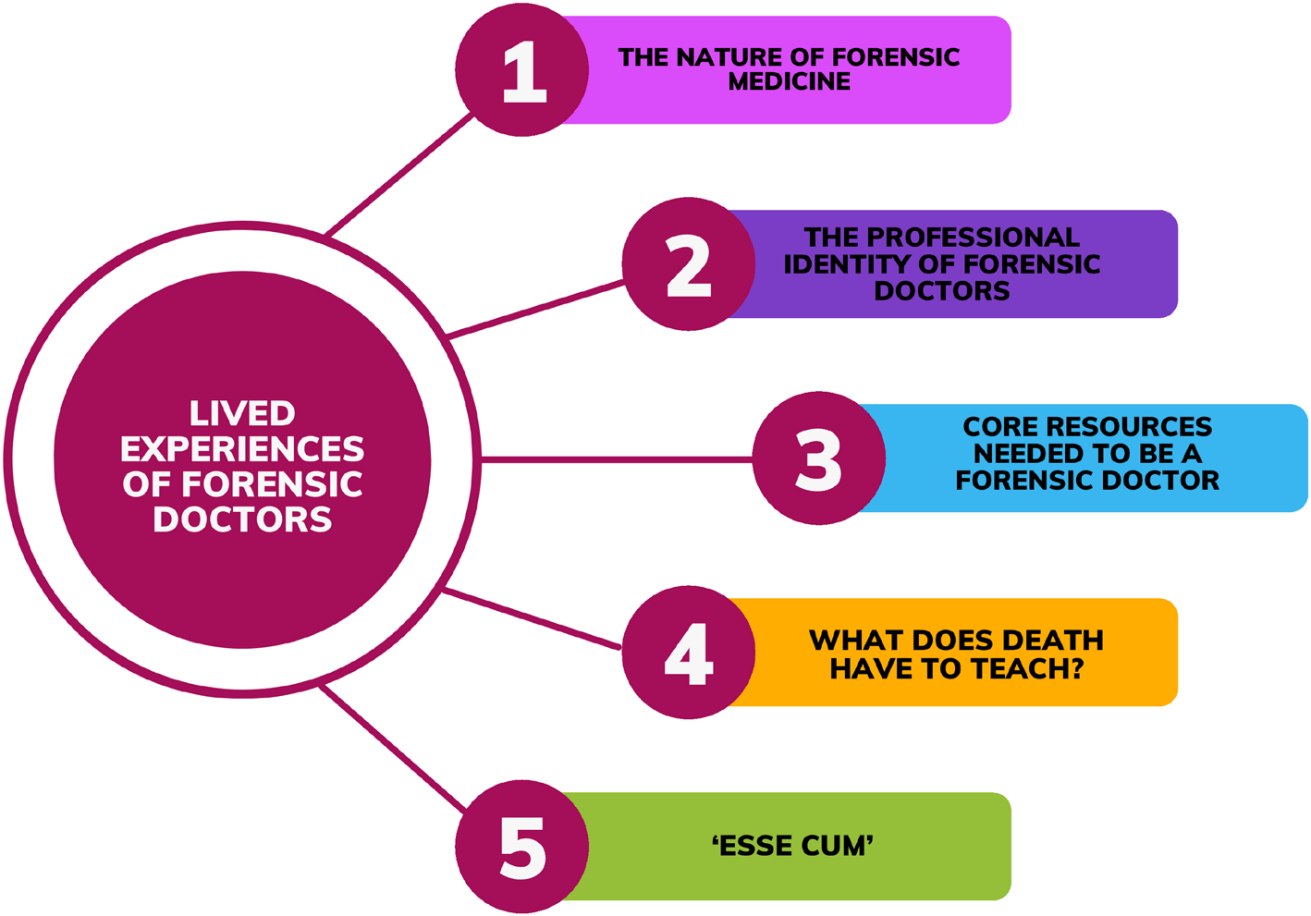
Themes from the in‐depth analysis.

Five main themes emerged from the in‐depth analysis as reported in Figure 1:

### 3.1 Theme 1: The nature of forensic medicine

The participants described the discipline of forensic medicine through four distinct sub‐themes which describe metaphors used to depict their professional experience: (1) Forensic Medicine as “Art,” (2) Forensic Medicine as an “Uncovering,” (3) Forensic Medicine as “Curiosity Unveiled,” and (4) Forensic Medicine as a “Connection.”

In describing forensic medicine as “Art,” participants compared their work to a theatrical performance or a symphony. They viewed the process of performing autopsies and conducting investigations as a complex and captivating masterpiece, requiring a coordinated effort among various individuals to create an experience that engages multiple senses:

In my opinion, it is comparable to a theater. The autopsy is performed in a tiered anatomical room, where residents can act as spectators. It is like an orchestra of people working together. There are very intense colors, mostly yellow and red, when the work is being done. The scenes are also very film‐like, such as going to the prosecutor's office to receive assignments, dealing with law enforcement, which can be extremely challenging, and then discussing cases in court if they go to trial. Even that feels theatrical and cinematic. There is also the odor—it's part of the scene. By following guidelines, even during the autopsy, we use the gastric content we collect, which adds to the smell. The smell is indeed part of it (P01, male, 1 year of experience). In my opinion, it is comparable to a theater. The autopsy is performed in a tiered anatomical room, where residents can act as spectators. It is like an orchestra of people working together. There are very intense colors, mostly yellow and red, when the work is being done. The scenes are also very film‐like, such as going to the prosecutor's office to receive assignments, dealing with law enforcement, which can be extremely challenging, and then discussing cases in court if they go to trial. Even that feels theatrical and cinematic. There is also the odor—it's part of the scene. By following guidelines, even during the autopsy, we use the gastric content we collect, which adds to the smell. The smell is indeed part of it (P01, male, 1 year of experience).

Another participant stated:

Certainly, it is like a symphony, with each phase of our work forming part of a larger composition. From receiving a call—sometimes in the middle of the night—and performing a site inspection in challenging conditions, to writing reports, obtaining formal assignments in court, and conducting autopsies, each step flows into the next. The process continues with sample collection, histological examinations, and laboratory tests. Throughout, we must document everything meticulously with writing and photographs. Finally, all the objective data are analyzed and interpreted in a medical‐legal discussion, leading to conclusions and answers to the posed questions (P03, female, 25 years of experience).

The second group of metaphors, *Forensic Medicine as an “Uncovering,”* was conveyed by participants through concepts, such as a puzzle, an investigator, and the phrase “bringing up the rear.” Participants emphasized that their role involves meticulous discovery work, where nothing is considered trivial in criminal or civil investigations. The metaphor of “bringing up the rear” underscores the reactive nature of their work, as highlighted by one participant: *“We are called when an event, often traumatic, has already happened”* (P10, female, 4 years of experience). Upon arriving at the crime scene, the investigative process begins, embodying the notion of forensic medicine as an enigma, as it represents:

A puzzle with many pieces, because it's a job with countless nuances. Sometimes it's very complex and difficult to piece together, but like a puzzle, the pieces eventually come together to form a complete picture. This is what fascinates me about the job—it offers new challenges every day, even though there is always a common thread connecting all the parts and pieces (P02, female, 3 years of experience).

The forensic doctor takes on the role of “an investigator, because you have to start from a place, from a mystery—such as the cause of death—and then, through various analyses, piece everything together to understand why and how that person died” (P11, female, 6 years of experience).

In the forensic investigative process, participants emphasized the importance of being open‐minded, flexible, and willing to look beyond the obvious, which they described through the metaphor of *“Curiosity Unveiled.”* They explained that, in some cases, the solution to a case is not immediately apparent, requiring them to “dig deeper.” As one doctor elaborated:

Curiosity about the end. Regardless of the case, we are faced with a body that cannot tell us what happened. Our task is to evaluate the events leading up to the death, which is inherently stimulating and fuels our curiosity. It's this curiosity about the end that defines our work (P07, female, 19 years of experience).

Their routine was described as both curious and peculiar, as it places them in contact with two worlds—the living and the dead. In this sense, being a forensic doctor was perceived as occupying a precarious position between these two realms:

A tightrope walker balancing between the living and the dead, between problems and their resolution… It's a strange world. We answer questions that only we can, marking the end of the story. When everyone else hasn't found a solution, we are the final phase, bridging what is no longer there and what still is (P08, female, five years of experience).

The fourth group of metaphors, centered on the unique position between life and death, was described as a “Connection.” Forensic doctors perceive themselves as being in a “Connection,” not only with the legal system but also with the families of the deceased:

Before starting the autopsy, we speak with the families of the deceased. We have a room with a table where we sit on one side, and they sit on the other. The image that comes to mind is this table where we interact with the families. This is where we learn about the deceased—when they were last seen, their illnesses, and in the case of suicides, their mental state in the final days. This interaction represents the core of our work (P12, male, 5 years of experience).

The forensic medicine has also been compared with a connection to various strands of essential scientific knowledge required for their work to the roots of a tree, emphasizing the foundational and interconnected nature of their expertise:

An image comes to mind: a tree with roots. These roots represent essential medical knowledge, but the work of a forensic doctor branches out into many areas, expanding further while still staying connected to its roots (P09, female, 14 years of experience).

The doctors' connection with life and death was described as transformative, promoting personal growth through their exposure to and engagement with human suffering:

It's like from dawn to dusk. Much of my work is at the sunset of life, but the dawn represents the experiences I've gained that continue to inspire me. It's a constant renewal, just like each day renews itself and is symbolized by dawn. That's why I used this metaphor. The dusk can be positive, but there are also negative aspects—not so much for me personally, but for the situations I inevitably face, especially not with the body itself, but with what surrounds death, particularly the pain felt by the relatives (P06, male, 38 years of experience).

### 3.2 Theme 2: The professional identity of forensic doctors

This theme highlights the unique professional role of forensic doctors, which involves a distinct form of caring. Unlike other medical specialties focused on healing, forensic doctors care for individuals (i.e., the deceased) who cannot speak for themselves, as well as for their grieving families. One participant explained:

In short, our professional purpose is not therapeutic; it is focused on assessment. Our patients are not individuals we care for continuously, but rather, we evaluate them from a forensic medical perspective in a single encounter. The goal is not therapeutic but different (P09, female, 14 years of experience).

Another participant stated:

In a way, we help people, not therapeutically, as we don't provide a cure for diseases, but by performing autopsies, we often help the family—more than the deceased—achieve justice. It's about helping the family or those seeking to understand what happened. We maintain a detached perspective, both medically and legally, on the cause of death, whether it's an illness or a homicide. Even though we don't treat diseases, we still provide a form of care, especially for the family (P12, male, 5 years of experience).

The sense of caring extended beyond the immediate outcomes of an autopsy. Participants expressed a profound sense of responsibility, particularly in terms of prevention, toward the deceased person's relatives:

In my experience, I have become particularly attached to cases of sudden cardiac death in young people. In these cases, we investigate whether the death was caused by a genetic condition, so we can inform the family about the potential risk for future children or relatives. From there, we begin the investigation, which may involve genetic tests or cardiological exams. If a genetic condition is found, it can be treated to prevent further incidents. This is where our role becomes crucial (P04, male, 5 years of experience).

Moreover, the participants emphasized a profound commitment to uncovering the “truth”:

Seeking the truth, both legally and biologically, is something that intrigues and stimulates me. Understanding why a person has died is a key aspect of this. Additionally, providing tools to those in the justice system, such as public prosecutors and judges, to help them reach legal truth in cases is also an important part of my work (P06, male, 38 years of experience).

And ensuring justice for the deceased:

Besides our offices, we spend a lot of time attending hearings at the courts and judicial offices because, ultimately, we are doctors who serve justice. Forensic medicine is medicine at the service of justice. I know that my work carries a certain responsibility, and this motivates me every day to do well, as I understand that everything I write has a direct impact on people (P03, female, 25 years of experience).

This sense of justice was reflected in providing relief to families seeking answers about what happened to their loved ones:

When you perform an examination and reach a diagnosis, that explains to the family the cause of death of their loved one, you see that they are more at peace, in a way, because you have given them an answer that they might not have had (P10, female, 4 years of experience).

Professional identity also encompasses an awareness of personal limitations, particularly in cases where reaching a definitive diagnosis is not possible:

In many cases, we are not able to determine what happened, but we have done everything possible to try to understand. And so, in my opinion, being able to be useful, even for those who remain, is essentially important (P12, male, 5 years of experience).

According to the participants, certain cases, such as homicide‐suicides, are particularly complex and challenging to investigate, consequently, a few cases might not have closure:

And it's not always possible to have an answer instantly because often it's necessary to process data, study it, and think it through […] it can happen, not so rarely, to be unable to reach an answer, and therefore to learn, um… to live with the fact that some situations cannot be framed or resolved […] There is no answer for everything (P02, female, 3 years of experience).

### 3.3 Theme 3: Core resources needed to be a forensic doctor

This theme highlighted the pivotal role of mentorship in navigating the complexities of forensic practice, acquiring the specialized skills required for the field, and managing the unique challenges inherent to the profession.

Participants expressed varying perspectives on the role of mentors. For some, the mentor was a role model—occasionally perceived as “God‐like”—who significantly influenced their decision to pursue a career in forensic medicine:

My mentor… my teacher […] When I first saw him… when I first met him, it was in the early 90s (laughs). Of the last century, the last millennium (laughs). I was fascinated by how he did his work; it captivated me. And then, his humanity in how he approached his work deeply moved me. I was fortunate to follow him and be his disciple […] (P03, female, 25 years of experience).

For others, the mentor contributed to a more challenging work environment, fostering hostility and, in some cases, fueling a desire to leave the profession:

We had a very hard time with the previous professor; the relationship wasn't good, and the environment wasn't good when I was a resident. Since the situation changed quickly, I decided to stay. Otherwise, I would have left because I was completely burnt out, you could say (P11, female, six years of experience).

Participants described the early years of residency as particularly challenging, especially as they struggled to adapt to a lack of support from their mentors:

My first crime scene investigation was with my professor. We arrived at the scene of a suicide, and I asked him, ‘Okay, professor, what should I do?’ He didn't answer, so I peeked at his notes, but he took the notebook away and said, ‘You do your job, and I'll do mine.’ I just stood there, and then he asked the technician for advice. My first year was very difficult (P04, male, five years of experience).

Others reflected on how their first year involved navigating challenging dynamics with their mentor, including experiences of authoritative behavior:

For several years, I had a mentor who was my professor… well, when we started, he wasn't yet a professor, but he taught me a lot. Our relationship was very complex and quite overbearing towards me […]. He was a textbook pathological narcissist. I was his favorite victim (laughs), meaning he felt very comfortable with me because I projected his thoughts. This relationship cost me a lot, but it also taught me a lot. However, in the end, it cost me more than it cost. During those years, I lost myself completely to work; I had a private life, but I struggled to find personal space because everything was directed towards doing what [Professor's name] wanted (P04, female, 14 years of experience).

Participants noted that managing both relational challenges and professional responsibilities requires a diverse set of competencies. Among these, rigorous technical and scientific skills were the most frequently cited:

I would definitely say anatomy, as a major area of study, along with physiology and pathophysiology. These are the fundamental subjects that provide the essential groundwork for completing death certificates and understanding how to correlate what you observe macroscopically with the underlying phenomena that led to death (P07, female, 19 years of experience).

Furthermore, participants emphasized the importance of being open to possibilities, as nothing is straightforward in forensic medicine. One participant explained:

Open‐mindedness is essential because evaluating cases requires flexibility and openness. Curiosity and the desire to learn should be at the forefront. I would place curiosity first—a love for knowledge. Scientific curiosity, which translates into enthusiasm for what you'll discover each day. In forensic medicine, every day is a discovery. With every case, whether it's an autopsy, you never know what you'll find; it's always like opening a box, and whatever happens, happens (laughs). Curiosity is fundamental—if you're not driven by curiosity, you won't appreciate it. Some people prioritize security or conservatism and may struggle to embrace new things, so this job wouldn't be suited for them, as every day is different (P04, female, 14 years of experience).

In other words, participants emphasized that superficiality has no place in their role. Once an investigation begins, it requires a sustained sense of curiosity and determination to see it through to its conclusion.

Another essential competency described by participants was the ability to find “the right thing to say” when interacting with family members. In forensic medicine, where traumatic deaths are common, the ability to engage sensitively and communicate effectively with grieving relatives was considered crucial.

For the living, there's the issue of making contact because there are situations where you can't just say, ‘I understand your situation’ since it's unrealistic to tell someone who has been held captive, ‘I understand’. Establishing a relationship with them is very difficult, in my opinion, because as a forensic doctor, you must act in a certain way. Your role as a doctor is simply to help them document their condition and certify the fact that they have suffered violence (P12, male, 5 years of experience).

Furthermore, participants acknowledged that engaging with bereaved family members could be one of the most challenging aspects of the job. It demanded specific skills to navigate the family's emotions, respect their wishes, and honor their rituals:

So, the most difficult part by far is dealing with the families. You see the families before the autopsy, exclusively before the autopsy, because there is a law that requires them to do what is called cadaveric identification, which I find extremely cruel and unnecessary. I mean, they might have already identified the body at the time of discovery, but they are called back to the morgue, which is certainly not a pleasant place to visit, and they are shown the body before any procedures are done. So, if it's [the body] covered in blood, it's covered in blood; if it has lacerations, it has lacerations. They have to say yes or no, it's him or it's not him, and they can't touch the body. It's a very quick process (P01, male, 1 year of experience).

Guiding a family through the process of confirming the identity of the deceased requires profound understanding and respect, particularly when complicated grief is involved:

In fact, there are family members who, in their pain, might… well, they might even lash out at the forensic pathologist who has to perform an autopsy. They might say something like, ‘What, after everything that's happened, you still have to come and make things worse?’ But of course, at that point […] there's a certain sensitivity that comes with being a person (P03, female, 25 years of experience).

Participants reflected on how being a forensic doctor meant they may be present in every parent's nightmare, the death of their child:

‘Especially with children, it's tough. Sometimes you have to go do an autopsy on a child, and even though I can do it, I have to convince myself that I can, you know? It's hard, but I tell myself it's my job, I must do it, so I do. But I already go into it feeling this way, and then maybe I find, I don't know, the teddy bear that the mother left behind… And it makes me cry [cries], sorry. Even before, when I imagine the suffering that mother must have gone through … [breathes deeply], it seems like something impossible to handle, even for me’ (P07, female, 19 years of experience).

These situations were particularly challenging to navigate because, as one participant explained, ‘Fundamentally, you never find the right thing to say. Every response seems either trivial or inappropriate’ (P02, female, 3 years of experience). Recognizing that spirituality and religion could provide comfort to grieving relatives, participants emphasized the importance of respecting and, where possible, accommodating the family's rituals:

For example, this morning I performed an autopsy […] he [the patient] had a small holy card with him, and we did everything we could to find it. By doing that, I was able to give some comfort to the family, who cared deeply about it. It cost me little to do it, and it was something that gratified me. They really cared about it. These may seem like trivial things, but they need to be valued to maintain a good relationship with the family and to be at peace with oneself. (P06, male, 38 years of experience).

The empathy and respect for the relatives’ beliefs were also reported by a further participant who stated:

Personally, I've looked into the beliefs of different regions regarding the desecration of the body, such as in Judaism, where the body is supposed to be buried as a whole. For instance, if there is an amputation, the amputated part is preserved and then buried together with the body. What I do is explain that I can't avoid taking samples because the law requires it. However, I assure them that the procedure will be minimized, limited strictly to what is necessary, out of respect for their beliefs. I do everything I can within the limits of the law. (P01, male, 1 year of experience).

Lastly, completing the cadaver's examination and arriving at a diagnosis was regarded as an essential aspect of providing comfort to grieving families. As one participant explained, ‘When you conduct a diagnostic autopsy and explain to the family the cause of their loved one's death, you can see that they become more at ease, in a way, because you've provided them with an answer they didn't have before’ (P10, female, 4 years of experience).

For forensic doctors, impartiality was identified as a vital skill—not only in approaching cases objectively but also as a coping mechanism to sustain their ability to perform this challenging and unique work.

‘But I think… it's really an egotistical point of view, given that these are not experiences I've had personally. I feel quite detached; I don't feel they are mine, and I don't qualitatively perceive them. I feel quite external, meaning I simply use the methodology and apply it to the case. It's clear that if someone is, undoubtedly guilty, I can't make something up. What someone else might do is purely follow a procedural standpoint’ (P01, male, 1 year of experience).

Regarding formal coping strategies, participants engaged in a variety of activities to manage the challenges of their work, such as seeking solace in beauty through art, nature, or sports. However, the most commonly reported coping strategy was avoidance and detachment. One participant reflected:

‘So, apart from specific cases that leave a lasting impression, when I arrive at the scene, whether it's at the site or in the autopsy room, for me, it's just work. I sort of block everything else out… all the surrounding context and focus on what I have to do at that moment. It's about blocking out the context’ (P10, female, 4 years of experience)

Another participant stated:

Lying still on that table [the corpse], I can't perceive them as people, because in the end, they don't talk, they don't complain, and they have a color that's not the same as the rest of us who talk and complain about life (laughs). So, it's as if only the shell of the person remains, and the personality, the things they liked, and the things they did seem like completely different matters. But this is something I've always thought about, really’ (P08, female, 5 years of experience).

Interestingly, participants with more years of experience noted that their ability to maintain detachment had been affected by their personal life circumstances. For instance, one participant reflected on how performing autopsies on children had become particularly challenging after becoming a parent:

Before having my child, I was less affected by death and could easily move on, accepting it as part of the job. However, after becoming a parent, the idea of death, especially my own, now scares me more, making me more sensitive. Emotionally, I've had stronger reactions, particularly when relatives identify their loved ones. It's difficult to maintain detachment in those situations, and I no longer feel able to perform autopsies on children (P09, female, 14 years of experience).

Some referred to how being detached was only possible by focusing on the procedure and technicality of the work being undertaken:

And then you focus more, for example, on studying anatomy. We perform the autopsy in anatomical planes, so first you remove the skin, then you isolate the muscle and examine it, noting things like, ‘Oh, look, the muscle is more or less developed, the presence of fat’ and then you teach the younger ones, so to speak. You pay close attention there because if you use the scalpel incorrectly, you risk cutting something you shouldn't. So you concentrate more on the procedure, on the act (P04, male, 5 years of experience)

Another coping strategy was social support with other forensic doctors. Participants considered that relatives and friends who were not from this profession might not understand what they must endure:

I find greater support from colleagues because, given that it's such a specific activity, it's probably difficult for those who don't practice it to fully understand it. (P02, female, 3 years of working experience).

### 3.4 Theme 4: What does death have to teach?

In this theme, the participants mentioned the lessons learned from constantly interacting with death‐related situations and how it influenced their perceptions of being alive.

Being constantly in contact with death taught the participants how to think about themselves and their way of living. The participants shared how the traumatic events they investigated stimulated them to *“measure”* and *“weigh”* the relevance of what they encountered in their own life‐world:

This work, dealing with often desperate cases and facing death daily, makes us see things not necessarily with the right perspective, but with the right measure. It helps us put things into perspective and not complain about trivial matters. It allows us to give the proper weight to things, realize how fortunate we truly are, be grateful, and not take anything for granted (P03, female, 25 years of experience).

Another participant echoed the sentiment of gratitude reflected in the quote above by stating:

It has always been something that made me feel very alive because you are surrounded by death, and it makes you feel your own life even more (laughs). You feel fortunate to be alive and not take for granted many things that people usually do because it's not every day that you remember you are alive unless you see someone who is dead (P09, female, 14 years of experience).

An awareness of human mortality and the finitude of existence led the participants to be more authentic and spontaneous in their lives:

In short, death has mainly influenced me in the sense that when I'm unsure about doing something, I say yes. It has made me value my private life more. If I can do something now, I try to do it, even if it makes my schedule harder. For example, if I can get something done this year instead of next, I'll do it (P01, male, 1 year of experience).

Another participant stated: “The possibility of dying at any moment sometimes makes me say, ‘live life and make the most of every moment because it could happen at any time’… you never know” (P06, male, 38 years of experience).

The participants' heightened awareness of death inspired a desire to strengthen their connections with loved ones. As one participant shared, “For example, in interpersonal relationships, even asking one more question about how a friend is doing, things like that, it's always useful to do so” (P04, male, 5 years of experience). Another participant reflected, “I started going out more and spending more time with my friends, dedicating much more time to myself even though I have less free time” (P11, female, 6 years of experience).

Nevertheless, exposure to death inevitably led to personal changes. Participants expressed concern that, as death became a routine part of their work, there was a risk of it being trivialized. They emphasized that death and dying are profound and extraordinary events that must always be approached with respect and dignity:

The risk is that one might lose sight of the fact that, even after seeing your thousandth deceased person, they are still an individual who has left behind a family suffering just as much as with the first case you encountered (P12, male, 5 years of experience).

Participants believed that any case is unique, and the deceased's dignity and their relatives' pain must not be taken for granted:

I could say that some might think—though it's not my case—that someone could become desensitized to death and treat it like just another job. While I can perform the work when I'm on duty, I can't handle the emotional aspects surrounding it. The risk, I believe, is that someone might lose sight of the suffering experienced by the relatives, such as a mother or child. This is a potential risk: failing to recognize death as a profoundly difficult event for the family and loved ones of the deceased. Perhaps the worst risk is treating the deceased as if they were just an object or a box (P07, female, 19 years of experience).

Accepting death as a natural part of life, participants shared their reflections on their own mortality. One participant observed, “I think I've already seen a lot of people who died for many reasons, so I have come to understand that one can die at any moment” (P11, female, 6 years of experience). Moreover, their constant exposure to death fostered a deeper appreciation for the beauty of living:

I am more at peace with the idea of dying because I believe it's a topic that's often avoided and treated like a taboo in our society. Sometimes, talking about death feels almost obscene. However, through this work, by being close to death and confronting it directly, I've come to understand it better and now face it more calmly. It's not that I'm not scared—there's still an irrational fear, but overall, I feel more at peace with the naturalness of it (P02, female, 3 years of experience).

The rationality of human finitude had limits, for instance, fearing death was mentioned by the participants, especially those who were parents:

Let's say that after I had my child, I started to ruminate much more because the idea of death scares me more. Before, the thought of my own death didn't worry me much, in the sense that no one experiences their own death because it's the others who live through it. But now that I have a small child, the idea of dying scares me quite a lot, so that's why I'm more sensitive to it. (P09, female, 14 years of experience).

Another participant reflected on how their fears of death had been transmitted to their children:

The other thing is that I realize I'm a bit stricter with my children because of this fear [death]—I've passed it on to them. The little one doesn't walk yet, but the other one does, and he becomes alarmed as soon as he sees a car approaching. I call him, [name], come here right now, hold on to Mommy.' So he does it, but you can see that he's terrified. This bothers me a bit because, in reality, it's a bit too much. But… I'm like this because, unfortunately, in my job, I see death, so with my children, at least, I'm a bit anxious about things like crossing the street. (P07, female, 19 years of experience).

### 3.5 Theme 5: “Esse cum”

“Esse cum,” a Latin term meaning “being with,” encapsulates the challenges, stigma, and stereotypes forensic doctors face in their relationships with others and society. This theme is divided into three sub‐themes: (1) forensic doctors and law enforcers, (2) living patients, and (3) societal imagination.

In the first sub‐theme, interactions with law enforcers, such as police officers and public defenders, were described as particularly stressful, especially for less experienced participants. These challenges were often attributed to a lack of respect and trust between forensic doctors and law enforcement. One participant shared:

One challenge is overcoming the distrust from those around you, whether it's the police, carabinieri, or magistrates. I think that's the biggest challenge so far, especially since I've just started. Also, as we mentioned, I haven't faced an actual trial yet and haven't testified. I think that will be the biggest challenge because I've observed cases with my professor—I was in the audience, so to speak—and you're really grilled, it's like being under interrogation. I believe that will be a significant test of… great stress, but I haven't experienced it yet (P10, female, 4 years of experience).

Participants also reflected on the challenges of working alongside police officers at crime scenes. They noted that these difficulties often stemmed from “speaking different languages,” which complicated the forensic investigation, particularly when police officers lacked a clear understanding of the doctor's role:

Sometimes you encounter an officer who says, ‘No, but the other one used to do it this way.’ I don't care how the other person did it, I do it my way. Then some officers might say, ‘No, you can't see the house, just handle the body.’ It becomes a standoff where you're trying to explain why you need to examine everything, like medications or anything else that might be useful, while they remain very focused on their specific duties (P04, male, 5 years of experience).

A further participant reflected:

There are situations where I want to speak with the prosecutor, as they requested my site visit, but the police officer prevents me from doing so, misrepresents what I say, and then walks away to provide their own account. I find that unprofessional (P05, female, 5 years of experience)

Participants noted that the lack of control over who was present and what was happening at the crime scene significantly hindered their ability to perform their job effectively:

The crime scene was a disaster. Despite it being a crime scene, people were everywhere—family, neighbors, even the secretary and a friend. One police officer was allowing people to enter and decide who could be there. I was trying to photograph the scene and had to ask people to move, but they were very uncooperative. When we examined the body, one of them, a lawyer, insisted on being present (P04, male, 5 years of experience).

This lack of control was further exacerbated by the law enforcers' insufficient empathy. One participant stated:

In my opinion, sometimes law enforcers lack sensitivity towards the grieving family. For instance, they sometimes show the photographic dossier of a corpse's injuries in front of family members, which should really be avoided. They have no tact whatsoever. It's especially problematic when the family is already in distress. I often see law enforcement handling these situations very poorly (P01, male, 1 year of experience).

The young female participants also shared a feeling of being harassed by law enforcers due to their gender:

I've noticed that being a young woman, law enforcers sometimes have difficulty, especially at the beginning. When you arrive, they might look at you for a moment—there's a bit of skepticism. However, in reality, they've never had any issues with my work because we know how to do our job. Still, because you're a woman and young, they do look at you a bit differently (P10, female, 4 years of experience).

Participants frequently expressed concerns about the expectations of law enforcers, such as public defenders, who not only demanded definitive answers but often took the forensic doctors' work for granted. This dynamic made it particularly challenging for forensic doctors to navigate interactions with public defenders, especially when their investigations yielded inconclusive results:

I would like public defenders to understand that ours is a difficult job that doesn't always yield clear answers. Unfortunately, there are times when we cannot address certain aspects that magistrates might want clarified. We may reach a certain point in our investigation but can't always meet their specific demands. I would tell them to recognize our limitations, to understand that the work is challenging, and that the answers we provide may not always align with their expectations. This is often hard for magistrates to accept (P07, female, 19 years of experience).

Stigma emerged as a recurring theme frequently emphasized by participants. They expressed that both society and medical colleagues often hold distorted and predominantly negative perceptions of their profession. These misconceptions, largely influenced by portrayals in TV series, were seen as reinforcing the image of forensic doctors as outliers or marginalized professionals. One participant remarked:

I would like to receive more respect—not just economically, but also personally—from magistrates, who sometimes view our work as something akin to butchery (P07, female, 19 years of experience).

Participants felt that stereotypes had devalued their professional work, with society often perceiving their occupation as odd or unsettling—“[those] weird people working with death” (P08, female, 5 years of experience). Another participant added:

Recently, there's been too much media attention on my profession, and I don't like it at all. It's being used for publicity on TV and in newspapers, which is an aspect I strongly dislike. I wish people understood the true importance of our work—not for the sensationalism but for its value in uncovering the truth and providing information that can assist clinicians in diagnostic reflection. I would prefer the scientific contributions of forensic medicine to be more widely recognized, rather than the harmful media focus (P06, male, 38 years of experience).

Participants felt that TV shows perpetuated the unrealistic expectation that forensic investigations always yield solutions—and do so within an unreasonably short time frame:

Sometimes they [law enforcers or families] want immediate answers like, ‘What happened? When did it happen? Why did it happen?’ But it's not like in the movies. It's important to convey that we are not super scientists who can solve everything—some questions remain unanswered. The job shouldn't provoke a ‘wow, how cool’ reaction as seen in films. Instead, it should be understood for what it is: a meaningful and interesting job, but not the sensationalized version portrayed in cinema (P02, female, 3 years of experience).

Beyond the misconceptions portrayed in TV shows, participants identified another factor that perpetuates negative stereotypes about forensic doctors: the undervaluation of working with death compared with other medical professions in Western society. Participants believed that contemporary society glorifies pro‐life strategies and often refuses to accept death as a natural part of human existence. This cultural tendency toward death avoidance reinforces the perception of death as less worthy of acknowledgment. As one participant explained:

Many times, we hear jokes like, ‘Oh, your patients don't complain’ or, ‘It must be easy for you since your patients don't complain!’ [laughs]. But it's definitely not like that. We have to manage a lot because the deceased comes with their own world, built by their loved ones (P03, female, 25 years of experience).

Another participant reflected:

The fact that they are deceased doesn't make the work we do any less important. It's a job that has significant public relevance. In my opinion, it's a serious job, even if I see many of my colleagues dismissing it and saying the opposite. It's not really like that. And I believe it's not well understood at all. There are aspects of forensic medicine that even clinicians don't know about (P01, male, 1 year of experience).

In addition to their work with the deceased, forensic doctors often interact with living patients. This aspect of their role was described as particularly arduous, as patients could sometimes behave arrogantly, demand falsified information, or act aggressively toward the doctor:

Some living patients can be challenging, as they often have strong opinions and believe they know more than the doctor, making it difficult to guide them. When I have to see ten patients in one afternoon, it can be quite exhausting, as many tend to be assertive, demanding, and confident in their own assumptions. It's not an aspect of the job I particularly enjoy (P07, female, 19 years of experience).

A further participant reported:

Being in contact with living people and their relatives daily is very stressful. People often look up information online, make their own diagnoses, and become more demanding. For instance, I once examined a man stabbed by his wife—thankfully not fatally—who asked me to lie in my report to protect her from prison. It put me in an uncomfortable position, but I still had to do my job (P10, female, 4 years of experience).

## 4 DISCUSSION

To our knowledge, this is the first qualitative study to explore the lived experiences of Italian forensic doctors using IPA. This methodological approach enabled the capture of complex experiential phenomena, offering insights into how exposure to death impacts personal and professional lives, professional identity, and interactions with justice and society. These findings contribute to a deeper understanding of the role of forensic doctors and inform the development of interventions aimed at reducing work‐related stress [16].

Our results highlighted the complexity of the forensic doctor's role, as articulated through metaphors, and underscored their impartiality and sense of justice as core components of the discipline. Professional identity emerged as a crucial factor for these doctors, connecting them to the tasks, responsibilities, values, and ethical standards that define their work [17]. This identity appears to serve as a protective factor, positively correlated with accepting attitudes toward death and negatively correlated with death anxiety [18]. These findings suggest that fostering a strong professional identity may enhance resilience and well‐being among forensic doctors, particularly in death‐related scenarios.

The study also emphasized how working in close proximity to death reshaped participants' perceptions of life, reminding them of the finitude of human existence and reinforcing the importance of spontaneity and interpersonal relationships. This aligns with existing literature suggesting that frequent contact with death can lead to a heightened sense of life's meaning, reduced death anxiety, and a shift toward non‐materialistic values [19, 20, 21]. Such closeness to death appears to prompt experiential changes, refocusing priorities, reducing rumination, and enhancing present‐moment awareness [21].

However, forensic doctors also face significant challenges, including societal misconceptions about their roles, stigma, and trivialization of their work. Stigma, which encompasses stereotypes, prejudice, and discrimination [22], has detrimental effects on mental health, increasing the risk of psychological distress, depression [23], and burnout [24]. The perceived undervaluation of their work by other professionals, such as law enforcers and clinicians, further exacerbates these challenges, potentially contributing to depression and anxiety [25]. Addressing these issues through public awareness and research is essential for mitigating stress, improving productivity [26], reducing burnout, and enhancing job satisfaction [27].

Positive mentorship was identified as a key factor in shaping the professional experiences of forensic doctors. While some participants recounted negative interactions with supervisors, the presence of a compassionate and professional mentor could counterbalance these experiences, fostering resilience, emotional support, and professional growth. This finding aligns with the broader literature on the role of mentorship in enhancing job satisfaction and reducing workplace stress [28]. Future interventions should emphasize the development and promotion of supportive mentorship programs within forensic medicine.

The theme “esse cum,” which explores the relational and societal dimensions of forensic doctors' work, revealed four distinct sub‐themes: interactions with law enforcers, relationships with living patients, societal imagination, and their complex interplay. These sub‐themes represent promising areas for future research. Investigations into interactions with law enforcers could identify barriers to effective collaboration, while analyses of societal perceptions might inform strategies to combat stigma and misconceptions about forensic professionals. Exploring these relational dynamics systematically could enhance understanding and guide the development of interventions to improve professional and personal well‐being.

Another avenue for future research is the exploration of personality typologies, mood and anxiety disorders, and their influence on forensic doctors' responses to professional challenges. While this study focused on lived experiences, incorporating personality, mood, and anxiety assessments in subsequent research could provide valuable insights into the interplay between individual traits, coping mechanisms, and vulnerability to mental health issues. Such findings would complement the current study by offering a more nuanced understanding of the factors contributing to stress and resilience.

Future research could also examine the alignment between forensic doctors' initial perceptions and expectations of their roles and the reality of their daily experiences. Understanding this dynamic could reveal gaps contributing to dissatisfaction or stress and identify strategies that enable these professionals to adapt and thrive. Insights from such studies could inform training programs designed to better prepare individuals for the realities of forensic work and support their long‐term professional development.

Additionally, incorporating structured peer support systems into forensic practice could address the emotional toll of the work. Facilitated process groups, peer mentoring programs, and informal support networks could provide safe spaces for sharing experiences, fostering emotional expression, and reducing feelings of isolation. Such resources could complement surveillance for mood and anxiety disorders, strengthening resilience and creating a more connected and supported workforce.

The participant's interactions with law enforcers and families of the deceased were characterized by professionalism and empathy. Despite the inherent stress, participants considered these interactions a fundamental part of their roles. Successfully managing these relationships and providing closure to grieving families was seen as a professional achievement. The desire for justice and the ability to support families in understanding their loved one's death contributed to a sense of purpose and improved job performance through positive work‐related identity, collective self‐esteem, and psychological capital [28].

Finally, our findings underscore the importance of fostering better communication between forensic doctors and law enforcers, as well as the need for emotional training to navigate family communication challenges. Addressing public misconceptions through education could further enhance the perceived value of forensic medicine. Death education programs for both professionals and the public have been shown to improve coping mechanisms and reduce death‐related anxiety, and they represent a valuable strategy for addressing these issues [29].

## 5 LIMITATIONS

This study has several limitations. First, our findings represent the professional life histories of only 12 forensic doctors, and while these narratives provide rich, in‐depth insights, they are not intended to be statistically generalizable to the entire population of forensic doctors in Italy. However, as emphasized in life‐history research, the goal is not to produce universally applicable conclusions, but to explore how professionals construct meaning from their career experiences. Future studies with larger and more diverse samples could expand upon these insights, potentially incorporating mixed‐method approaches to integrate qualitative and quantitative findings.

Second, our sampling strategy relied on professional networks, which may have introduced selection bias. While we aimed for diversity in terms of experience, gender, and work context, other factors, such as regional variation in forensic practice, were not systematically analyzed. Future research could use longitudinal or comparative life‐history studies to examine how career trajectories and resilience strategies evolve over time in different institutional and cultural contexts.

Lastly, our study is context‐specific, focusing on forensic doctors within the Italian healthcare and judicial systems. While some of our findings may resonate with forensic professionals in other countries, cultural and systemic differences must be considered. Future research comparing life histories of forensic doctors across different legal systems could provide valuable cross‐cultural insights into how professional identity is shaped by institutional and societal influences.

## 6 CONCLUSIONS

This study explored the lived experiences and life histories of 12 forensic doctors in Italy using the IPA methodology. Five key themes highlighted the unique challenges associated with constant exposure to suffering and death in their professional lives. These experiences profoundly influenced their personal and professional identities, as well as their interactions with others. The findings underscore how working closely with death inspired forensic doctors to value meaningful experiences, nurture personal relationships, and maintain a commitment to justice, professionalism, and compassion for bereaved families.

Additionally, this study sheds light on the significant impact of social stigma on the mental health of forensic doctors, which can exacerbate stress, depression, and burnout. Despite these adversities, participants exhibited resilience through positive coping strategies and reported a sense of job satisfaction. The themes identified in this study provide valuable insights for institutions and researchers seeking to design targeted interventions to support the mental health and well‐being of forensic doctors. Initiatives, such as peer support programs and communication training, could play a pivotal role in enhancing their professional quality of life.

Furthermore, the findings emphasize the importance of raising societal awareness about the critical role forensic doctors play in both healthcare and the justice system. By addressing misconceptions and promoting an understanding of their contributions, institutions can foster greater respect and support for these professionals.

## CONFLICT OF INTEREST STATEMENT

Each author declares that he or she has no commercial associations (e.g., consultancies, stock ownership, equity interest, and patent/licensing arrangement) that might pose a conflict of interest in connection with the submitted article.
